# A unified framework for interpretable elevator fault diagnosis and predictive maintenance via style-aware CoT fine-tuning

**DOI:** 10.1371/journal.pone.0353219

**Published:** 2026-07-07

**Authors:** Yuhao Wang, Junjie Huang, Qiang Zhang

**Affiliations:** 1 Zhejiang University, Hangzhou, China; 2 ZJU-Hangzhou Global Scientific and Technological Innovation Center, Hangzhou, China; Shanghai University, CHINA

## Abstract

Although Large Language Models (LLMs) have shown potential in industrial applications, they encounter significant hurdles in vertical scenarios like elevator maintenance, including hallucinations, lack of domain specificity, and an inability to interpret numerical physical states. To bridge this semantic-physical gap, this paper proposes a Unified Style-Aware Chain-of-Thought (SA-CoT) framework tailored for Small Language Models (SLMs). The novelty of our approach lies in two aspects: first, we construct a robust instruction dataset using a style-aware augmentation strategy to simulate diverse real-world user behaviors and noise; second, we innovate by textualizing raw sensor data, enabling the fine-tuned 4B-parameter SLM to generate high-dimensional embeddings for downstream numerical analysis. Experiments demonstrate a dual breakthrough: in generative diagnosis, the SA-CoT framework consistently outperforms general models, achieving a 5.6-fold improvement in BLEU-4 scores compared to GPT-4o. Furthermore, its embeddings capture physical features more effectively than traditional baselines, yielding highly competitive accuracy in Alarm Type Classification and Vibration Magnitude Regression. These results suggest that domain-aligned SLMs offer a robust and cost-effective framework for autonomous predictive maintenance, indicating that knowledge density plays a more critical role than parameter scale in specialized industrial applications.

## Introduction

With the acceleration of global urbanization, elevators have become the indispensable vertical transportation hubs of modern high-rise infrastructures. The safety and operational efficiency of these systems are of paramount importance; a single failure can lead not only to service interruptions and substantial economic losses but also to severe casualties. Traditionally, elevator maintenance relies heavily on the expertise of senior technicians, who diagnose faults based on fragmented technical manuals and personal experience. However, this human-dependent model is becoming unsustainable due to the increasing complexity of electromechanical systems and the shortage of qualified personnel. Consequently, there is an urgent demand for intelligent systems capable of automating fault diagnosis and predictive maintenance. The maintenance paradigm of elevator systems is undergoing a critical transition from reactive “breakdown maintenance” to proactive "Predictive Maintenance" (PdM). Driven by the rapid advancements in the Internet of Things (IoT) and Artificial Intelligence (AI), data-driven approaches have emerged as a dominant force in enhancing the perception and diagnostic capabilities of elevator systems.

In recent years, data-driven approaches—particularly Deep Learning (DL) and Computer Vision (CV)—have made significant strides in elevator safety. Such as detecting abnormal passenger behaviors [1], identifying electric bicycles [2], and segmenting control panels for robots [3]. However, these methods are inherently “surface-level”—they remain blind to internal electromechanical failures (e.g., circuit logic errors) that are invisible to cameras. Parallelly, signal-based methods utilize sensor data (e.g., vibration) to diagnose internal faults [4]. To mitigate the scarcity of fault data, techniques like Generative Adversarial Networks (GANs) have been introduced to synthesize realistic signals [5]. Others have explored Knowledge Graphs to structure maintenance logs [6]. Despite these successes, a critical “semantic gap” remains: most deep learning models operate as “black boxes.” They can output a fault code but fail to explain the reasoning process or provide actionable, step-by-step repair guidance derived from technical manuals.

The emergence of Large Language Models (LLMs) introduces a promising framework to bridge this gap, demonstrating remarkable potential in encoding knowledge and performing logical deduction. However, deploying general-purpose LLMs (e.g., GPT-4) in vertical industrial scenarios faces three critical hurdles. First, general models are prone to hallucinations, often fabricating plausible but incorrect technical details when lacking domain-specific constraints. Second, there exists a significant language-physical gap; standard LLMs are designed to process text but struggle to interpret the raw numerical sensor data (e.g., vibration acceleration, voltage) vital for establishing the physical state of equipment. Finally, the domain suffers from data scarcity, as high-quality, structured chain-of-thought (CoT) data for elevator maintenance is extremely rare compared to general internet text.

To address these challenges, this paper proposes a Unified Style-Aware Chain-of-Thought (SA-CoT) framework tailored for Small Language Models (SLMs), specifically leveraging the lightweight Qwen3-4B. Unlike previous studies that focus solely on either visual recognition or numerical classification, our approach integrates semantic reasoning with physical state perception. We introduce a novel data augmentation strategy that simulates diverse maintenance scenarios—ranging from novice descriptions to expert shorthand and noisy field inputs—to robustly align the model with real-world complexities. Furthermore, we innovate by textualizing raw sensor data, enabling the fine-tuned SLM to generate high-dimensional embeddings that serve as effective features for downstream numerical tasks. While this study focuses on elevator systems as a primary testbed, the proposed SA-CoT framework and the concept of textualized physical embeddings exhibit strong generalizability.

The main contributions of this paper are summarized as follows:

Style-Aware CoT Data Construction: We propose a robust instruction construction strategy. By applying style transfer to augment standard manuals into diverse linguistic patterns (e.g., novice descriptions, noise-injected queries), we significantly enhance the model’s ability to handle informal user inputs in real maintenance scenarios.

Deep Domain Alignment via SLMs: We fine-tune a lightweight 4B-parameter model (Qwen) using a rigorous “Source-Analysis-Conclusion” CoT strategy. This forces the model to ground its reasoning in proprietary documents, effectively reducing hallucinations and outperforming general baselines in source compliance.

Unified Representation Learning: Beyond generative diagnosis, we explore the latent representation capability of the fine-tuned model. We utilize the high-dimensional embeddings from the SLM to perform downstream numerical tasks, achieving state-of-the-art accuracy in Alarm Type Classification and Vibration Magnitude Regression. This validates that our framework serves as a versatile agent for both qualitative interpretation and quantitative prediction.

## Related works

### Computer vision for elevator safety

Computer Vision (CV) technologies have been extensively deployed for monitoring external elevator environments and ensuring passenger safety. Early works focused on standard object detection; for instance, Liu et al. [3] released a benchmark dataset for elevator button segmentation to facilitate robot-elevator interaction. Building on this, Choi et al. [7] proposed an attention-based DETR model to improve button detection accuracy under variable lighting conditions. To address safety hazards, Cao et al. [2] constructed a specialized dataset for identifying electric bicycles entering elevators, while Apaza Chullunquia et al. [8] implemented a lightweight SSD-MobileNet on embedded devices to detect unauthorized access in freight elevators. Furthermore, Lei et al. [1] utilized video classification to identify abnormal passenger behaviors, and Kim et al. [9] evaluated YOLOv8-based segmentation on NPU platforms for efficient indoor perception. Thakur et al. [10] also applied deep learning for defect analysis in additive manufacturing of elevator components. However, despite their efficacy in perceiving visible risks, these vision-based methods remain restricted to “surface-level” observation. They are inherently incapable of diagnosing internal electromechanical failures—such as circuit logic errors or hidden mechanical wear—that lie beyond the camera’s field of view.

### Data-driven fault diagnosis and predictive maintenance

In recent years, signal-based deep learning methods utilizing sensor telemetry (e.g., vibration, noise, and electrical current) have emerged as the mainstream paradigm for Predictive Maintenance (PdM) in industrial systems. A significant portion of existing research focuses on extracting complex patterns from specific mechanical subsystems. For instance, Wang et al. [11] proposed a convolutional recurrent network with time-frequency attention to process non-stationary elevator noise, while He et al. [12] utilized deep learning fitting combined with channel attention to diagnose traveling cable faults. To capture high-fidelity mechanical anomalies without the drawback of physical sensor degradation, recent advancements have introduced novel modalities, such as non-contact optical vibration sensing coupled with dual-branch deep learning [13]. Moreover, comprehensive intelligent PdM frameworks integrating multi-sensor fusion have been widely explored to enhance diagnostic robustness across complex mechanical environments [14,15].

Beyond feature extraction, considerable efforts have been dedicated to overcoming inherent algorithmic challenges, particularly data scarcity and domain shifts. To tackle the perennial issue of data imbalance in fault diagnosis, Lv et al. [5] successfully employed Generative Adversarial Networks (GANs) to synthesize realistic fault signals. In a similar vein, Pan et al. [4] leveraged transfer learning for elevator door system fault prediction, while Feng et al. [16] introduced unsupervised subdomain contrastive adaptation techniques to address domain shifts under varying operational conditions. Additionally, Wang et al. [17] developed a lightweight diagnostic model with enhanced temporal correlations to optimize computational efficiency.

Expanding from component-level diagnosis to system-level operations, research has also explored broader maintenance and control strategies. Notable examples include the Bootstrap-based life prediction method for traction systems introduced by Zhang et al. [18], and the hybrid approach combining imitation learning with reinforcement learning for efficient elevator dispatching proposed by Wan et al. [19].

However, despite achieving high predictive accuracy in specific tasks, these data-driven discriminative models fundamentally operate as “black boxes.” While they can effectively ingest numerical arrays to output fault probabilities or classification labels, they inherently lack semantic reasoning capabilities. Consequently, they are unable to explain the physical root causes behind a detected anomaly or synthesize actionable, step-by-step repair instructions grounded in technical manuals—a critical cognitive gap in real-world industrial maintenance.

### Large language models in industrial maintenance

The integration of Large Language Models (LLMs) marks a transformative era in industrial engineering by shifting paradigms from static, rule-based systems to dynamic, semantic-aware solutions. A recent comprehensive survey by Raza et al. [20] underscores this versatility across manufacturing sectors, emphasizing the capacity of LLMs to automate complex natural language processing tasks and synthesize technical insights. Within the specific domain of maintenance optimization, research trajectories have generally evolved into both human-assistive and autonomous frameworks. Exploring the assistive potential, Angelopoulos et al. [21] proposed an integrated architecture that combines LLMs with Augmented Reality to streamline technician workflows through real-time visual guidance. From a more autonomous perspective, Di Maggio [22] reviewed the emerging concept of agentic AI, advocating for a transition toward independent agents capable of closed-loop perception and reasoning. Advancing this autonomous vision, Sudhakara et al. [23] demonstrated that LLMs can effectively fuse heterogeneous data streams, such as historical maintenance logs and sensor readings, to generate contextualized diagnostics. This capability is further supported by recent studies integrating LLMs into multimodal anomaly detection and real-time Internet of Things stream processing [24,25]

Despite these significant advancements, current industrial maintenance paradigms exhibit critical limitations that the present work explicitly seeks to resolve. Traditional data-driven and deep learning methods [13,16,17] aggressively optimize numerical feature extraction but fundamentally operate as semantic black boxes, failing to provide the transparent, text-based reasoning required by field technicians. Conversely, contemporary LLM-based assistive systems [21,23] often remain decoupled from the raw physical sensor layer and rely on massive, computationally expensive foundational models that are highly susceptible to domain-specific hallucinations. The methodology proposed in this paper differentiates itself through a unified, hybrid approach that bridges this semantic-physical gap. By systematically textualizing raw sensor data, the framework enables a lightweight, edge-deployable Small Language Model (Qwen3-4B) to function simultaneously as an interpretable reasoning engine and a highly effective numerical feature extractor. Furthermore, by strictly aligning this model with a novel, style-augmented Chain-of-Thought dataset, the proposed framework achieves expert-level interpretability and robust predictive accuracy, effectively overcoming the prohibitive computational overhead and hallucination risks associated with massive foundational models.

## Method

**Fig 1:**
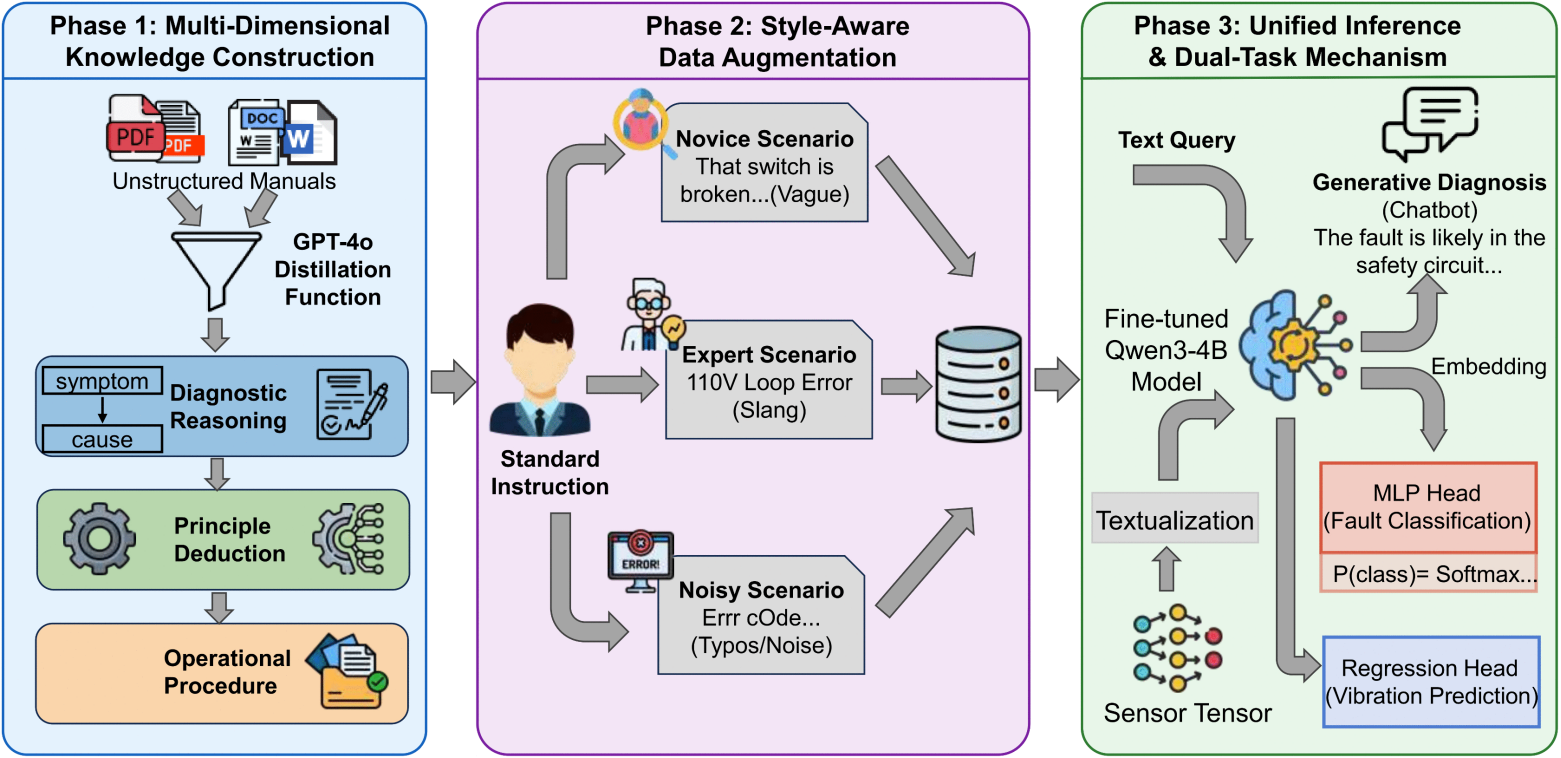
Schematic illustration of the proposed SA-CoT framework for industrial maintenance. The framework consists of three sequential modules designed to empower Small Language Models (SLMs) with expert-level reasoning. **Phase 1 (Left):** Unstructured technical manuals are converted into high-quality Chain-of-Thought (CoT) data. A distillation process categorizes knowledge into Diagnostic Reasoning, Principle Deduction, and Operational Procedures. **Phase 2 (Middle):** To address the distribution shift between formal manuals and field queries, a Style-Aware Augmentation module expands the seed dataset. A transfer function generates scenario-specific variants, including vague descriptions for novices, slang for expert, and corrupted text for noisy environments, forming a robust training corpus. **Phase 3 (Right):** The fine-tuned Qwen3-4B model utilizes a Dual-Task mechanism. It accepts both textual queries and serialized sensor tensors to output natural language diagnoses (Generative Head) and physical state predictions (Discriminative Head) via the embedding.

We propose a Unified Style-Aware Chain-of-Thought (SA-CoT) framework designed to empower Small Language Models (SLMs) with expert-level diagnostic reasoning and physical state perception capabilities. As illustrated in Fig 1, the framework consists of three distinct phases: (1) Multi-Dimensional Knowledge Construction, where unstructured manuals are converted into structured reasoning chains; (2) Style-Aware Data Augmentation, which simulates diverse user personas and environmental noise to align the training distribution with real-world scenarios; and (3) Dual-Task Fine-Tuning, which optimizes the Qwen3-4B model for both generative diagnosis and discriminative feature extraction.

### Multi-dimensional CoT data construction

Conventional instruction tuning datasets typically employ a superficial “Input-Output” (x→y) mapping. While effective for general chit-chat, this paradigm fails to bridge the cognitive gap required for industrial troubleshooting, where the path to a solution is often non-linear and necessitates multi-hop reasoning. To address this, we formalize the diagnostic process not as a direct mapping, but as a Chain-of-Thought (CoT) generation function $P(y|x)=\sum_{c}P(y|c,x)P(c|x)$, where *x* represents the fault query, *y* is the final solution, and $c=\{c_{1},c_{2},...,c_{n}\}$ denotes the latent sequence of reasoning steps.

To operationalize this mathematical formulation, we constructed a knowledge distillation pipeline leveraging GPT-4o to extract expert-level logic from proprietary maintenance manuals (encompassing Safety Circuit Schematics, Error Code Registers, and Field Maintenance SOPs). During this generation phase, the theoretical intermediate reasoning variables $c_{i}$ are deterministically instantiated through a mandatory JSON template structure. Rather than allowing unconstrained text generation, the latent sequence *c* is forced into discrete, observable sub-steps: *c*_1_ represents “Source Confirmation” (mandating the explicit citation of the proprietary manual filename), and *c*_2_ represents “Phenomenon Analysis” (the logical derivation of physical causality). The final diagnostic conclusion *y* is thus strictly conditioned on these explicit intermediate states, effectively approximating $P(y|c_{1},c_{2},x)$. By enforcing this structural rigor during data construction, the fine-tuned Small Language Model (SLM) is mathematically and practically constrained to replicate this exact deterministic reasoning pathway.

Through this highly constrained pipeline, the unstructured source documents were parsed and restructured into three distinct CoT archetypes. This diverse categorization ensures the model acquires a holistic, multi-dimensional understanding of the equipment:

- **Diagnostic Reasoning (**$D_{diag}$**):** This category models the abductive inference process used by senior technicians. Unlike simple pattern matching, the reasoning chain explicitly simulates hypothesis generation and verification: *Symptom Observation* (e.g., “E5 error”) → *Principle Alignment* (e.g., “Safety circuit open”) → *Exclusionary Check* (e.g., “Verify speed governor first”) → *Root Cause Localization*. This trains the model to prioritize high-probability causes before suggesting component replacement.
- **Principle Deduction (**$D_{prin}$**):** To prevent rote memorization, this category focuses on mechanistic interpretability (“White-box” understanding). The chain is required to explain the underlying electromechanical logic of a component (e.g., “How the Door Lock Loop interlock functions”) before deriving a solution. This ensures the model understands the physical causality behind a fault, enabling better generalization to unseen scenarios.
- **Operational Procedure (**$D_{oper}$**):** Focusing on safety compliance and execution, this category enforces strict adherence to industrial Standard Operating Procedures (SOPs). The reasoning chain is structured as a rigorous protocol: *Safety Confirmation* (e.g., “Switch to Inspection Mode”) → *Tool Preparation* (e.g., “Prepare multimeter”) → *Step-by-Step Execution* → *Post-fix Verification*. This instills a “Safety-First” bias in the model’s generation process.

### Style-aware data augmentation strategy

A critical impediment to deploying LLMs in industrial settings is the linguistic distribution shift between the canonical, well-structured language of training manuals and the heterogeneous, often fragmented queries encountered in real-world operations. To bridge this gap, we introduce a Style-Aware Augmentation Module that functions as a domain-adaptive style transfer mechanism. We define the augmentation process as a mapping function $T_{style}(I_{std},s)\to I^{\prime }$, where a standard seed instruction $I_{std}$ is transformed into a stylized variant $I^{\prime }$ conditioned on a target persona $s\in \{S_{novice},S_{expert},S_{noisy}\}$. This strategy expands our seed dataset into a robust corpus ${𝒟}_{train}$ by simulating three distinct operational realities:

- **The Novice Scenario (**$S_{novice}$**):** Targeting the Cognitive Gap. This style simulates interaction with property managers or non-technical personnel who lack domain vocabulary. The transformation $T(\cdot ,S_{novice})$ employs a Teacher LLM to rewrite instructions by replacing precise terminology with vague descriptors (e.g., converting “contactor” to “that black switch”). This forces the model to learn intent inference, training it to provide patient, step-by-step guidance despite ambiguous inputs.
- **The Expert Scenario (**$S_{expert}$**):** Targeting Communication Efficiency. This style emulates the high-context communication between senior technicians. The transformation $T(\cdot ,S_{expert})$ performs aggressive summarization and jargon substitution (e.g., “Safety Circuit” → “110V Loop,” “Error Code E4” → “E4”). Training on this subset adapts the model to professional shorthand, enabling it to function as a highly efficient assistant that skips basic explanations and delivers immediate, technical SOPs.
- **The Noisy Scenario (**$S_{noisy}$**):** Targeting Token-Level Robustness. To mimic the harsh reality of field input (often typed in a hurry or transcribed via voice-to-text), we apply synthetic noise injection. This includes random character deletion, phonetic typos (homophones), and subject omissions. Exposure to these perturbations trains the model to perform robust semantic reconstruction, ensuring that diagnostic accuracy remains high even when the input syntax is corrupted.

To ensure the reliability of the synthetically augmented dataset and prevent label contamination—particularly when introducing linguistic noise or novice phrasing—we implemented a rigorous, multi-tiered quality control pipeline. The most critical challenge in generating synthetic instructional data is the risk of the generative LLM hallucinating or injecting factual errors into the diagnostic reasoning. To completely eliminate this risk, we employed an *asymmetric augmentation strategy*. During the generation of the $S_{novice}$, $S_{expert}$, and $S_{noisy}$ variants, GPT-4o was strictly restricted to rewriting only the user query ($I_{std}$) to simulate the target persona. The corresponding Chain-of-Thought diagnostic response (*Output*) was deep-copied directly from the original, expert-derived seed dataset without any modification. This structural constraint guarantees that regardless of how informal or corrupted the input query becomes, the model is consistently trained to map it to a pristine, 100% factually accurate reasoning pathway. Furthermore, all generation processes were programmatically constrained to output valid JSON structures; a backend validation script automatically discarded any outputs containing parsing errors, missing fields, or empty responses to maintain strict structural integrity across the corpus.

### Fine-tuning of small language models

We employ Qwen3-4B, a lightweight yet capable transformer-based model, as our backbone. The choice of a 4B parameter scale strikes an optimal balance between reasoning capability and deployment feasibility on edge devices (e.g., industrial PCs). To align this generalist backbone with the specific requirements of fault diagnosis, we implement Parameter-Efficient Fine-Tuning using Low-Rank Adaptation (LoRA) within a Supervised Fine-Tuning (SFT) paradigm, utilizing our style-augmented corpus ${𝒟}_{train}$. The optimization process seeks to minimize the negative log-likelihood of the target Chain-of-Thought sequence given the stylized input prompt. The objective function ${ℒ}_{SFT}$ is defined as follows:


$$\begin{array}{c} {ℒ}_{SFT}=-\sum_{t=1}^{L}\log P(u_{t}|u_{<t},ℐ) \end{array}$$ (1)

where ℐ denotes the input instruction containing specific style markers, $u_{t}$ represents the *t*-th token of the ground-truth reasoning path, and *L* is the *t*otal sequence length. Through this supervised paradigm, the model effectively approximates a conditional probability distribution *P*(*y*|*x*, *s*). This formulation ensures that the model does not merely memorize answers but learns to implicitly recognize the stylistic features *s* embedded within the query *x* and dynamically modulate the complexity and tone of the response *y* to match the user’s expertise level.

### Unified inference for generative and numerical tasks

When integrating 1D continuous sensor telemetry with Large Language Models (LLMs), selecting the optimal input representation is critical. While conventional deep learning approaches often employ direct numerical embeddings—mapping scalar values into continuous dense vectors via linear projection layers—applying this paradigm directly to pre-trained LLMs introduces substantial challenges. Injecting non-textual continuous vectors disrupts the LLM’s native tokenization alignment and necessitates the training of an intermediary cross-modal projection layer. In data-constrained industrial scenarios, this approach is computationally expensive and risks catastrophic forgetting of the LLM’s pre-trained logical reasoning capabilities.

To circumvent these structural issues, we deliberately adopt a Textualization strategy, formalized as a serialization interface $\phi :{\mathbb{R}}^{n}\to 𝒯$. Instead of forcing continuous vectors into the embedding space, this function seamlessly projects a raw physical feature vector *v* into the LLM’s native vocabulary space as a structured natural language template. For instance, a numerical array is transformed into a context-rich descriptor: “Current speed is 0 m/s, cabin temperature is 28 degrees Celsius, x-axis acceleration is 0.02g.” Widely recognized in recent tabular-LLM research, this paradigm serves as a lightweight semantic bridge. It ensures that numerical states are processed by the LLM’s self-attention mechanisms in the exact same manner as text, allowing the model to leverage its robust pre-trained cognitive logic to infer relationships between physical variables and diagnostic outcomes without architectural modifications.

For the discriminative and regression downstream tasks, we freeze the weights of the fine-tuned Qwen3-4B backbone to preserve its learned domain alignment. We extract the high-dimensional embedding of the final token’s last hidden state, denoted as $h_{last}\in {\mathbb{R}}^{d}$, which serves as a compressed semantic summary of the equipment’s current physical status. To generate numerical predictions, we attach lightweight, task-specific projection heads to this frozen encoder. For Fault Classification, a Multi-Layer Perceptron (MLP) maps $h_{last}$ to a categorical probability distribution $P(class)=\text{Softmax}(W_{c}\cdot h_{last}+b_{c})$. For Vibration Regression, a linear layer estimates the scalar magnitude ${\hat{y}}_{vib}=W_{r}\cdot h_{last}+b_{r}$. This architectural design effectively validates our hypothesis that fine-tuning on domain-specific texts aligns the model’s latent semantic space with physical industrial realities, thereby significantly enhancing its representation capability for numerical analysis.

## Results

This section provides a comprehensive empirical evaluation of the proposed SA-CoT framework. The analysis is structured to address two pivotal research questions: First, whether a lightweight Small Language Model with 4B parameters, when subjected to style-aware fine-tuning, can outperform general-purpose foundational models like GPT-4o in specialized industrial diagnosis. Second, whether the semantic representations acquired by the SLM can serve as effective feature extractors to enhance performance in numerical downstream tasks, specifically fault classification and vibration regression.

### Experimental setup

To ensure full reproducibility, the detailed composition of our dataset is explicitly outlined. The foundational raw text was sourced from 11 proprietary technical libraries (e.g., “Practical Manual for Common Elevator Fault Maintenance,” “Safety Circuit Analysis”). To preserve continuous semantic context while optimizing the context window for GPT-4o, these documents were segmented using a chunk size of 1,500 words, resulting in 56 distinct document chunks. For each chunk, GPT-4o was prompted to distill 3–5 comprehensive Question-Answer pairs covering multiple dimensions (fault diagnosis, principle analysis, and operational procedures), yielding an initial Seed CoT Dataset of 240 high-quality pairs.

Following the style-aware augmentation pipeline, this seed dataset was expanded to encompass four distinct linguistic categories (the Standard seed, plus the Novice, Expert, and Noisy variants) in a strict 1:1:1:1 distribution ratio (240 pairs per style). Consequently, the final compiled dataset comprised 960 QA pairs. This corpus was randomly partitioned using a 9:1 split, yielding 864 training samples and 96 test samples. To rigorously prevent data leakage, manual cross-verification was conducted post-split to guarantee that no overlapping fault scenarios or identical queries existed between the training and testing sets.

All model fine-tuning and empirical evaluations were conducted on a hardware cluster equipped with four NVIDIA A100-SXM4 (80GB) GPUs. To demonstrate the inherent algorithmic robustness of the SA-CoT framework, default random seeds were retained without aggressive hyperparameter tuning. We employed Low-Rank Adaptation (LoRA) to efficiently fine-tune the Qwen3-4B backbone, targeting all linear modules with a rank of *r* = 8. The maximum sequence length was truncated at 2048 tokens. During the Supervised Fine-Tuning (SFT) stage, the model was trained for 30 epochs using a base learning rate of $1.0\times 10^{-4}$, coupled with a cosine learning rate scheduler and a 0.1 warmup ratio. The optimization process utilized a per-device training batch size of 4 and 8 gradient accumulation steps, with *bf16* mixed-precision enabled to significantly optimize the GPU memory footprint.

For fair baseline comparisons in the generative diagnosis task, all evaluated foundation models (e.g., zero-shot GPT-4o) were conditioned with an identical system prompt: *“You are an elevator maintenance expert. Please analyze the fault based on the user’s description and provide detailed troubleshooting steps.”* The user prompts were drawn directly from the 96 queries in the testing set. The exact training paradigms and textualization rules for the downstream numerical tasks are consistently maintained as described in their respective methodology sections.

To ensure the statistical reliability of our findings and mitigate the impact of randomness, we conducted rigorous statistical analyses across all experimental tasks. For the generative quality evaluation (Table 1), conducting multiple independent fine-tuning runs of the LLM was computationally prohibitive. Therefore, we employed a non-parametric Bootstrap Resampling approach to estimate the statistical variance. Specifically, we resampled the test set predictions with replacement 1,000 times to derive the mean, standard deviation (SD), and 95% confidence intervals (CI) for all metrics. For the downstream tasks, including Fault Classification and Vibration Prediction (Table 2), we conducted 4 independent training and evaluation runs, each utilizing a distinct random initialization seed. All quantitative results are now reported in the format of Mean ± SD. The consistently tight standard deviations observed across both the bootstrap simulations and the independent training runs provide strong empirical evidence that the performance enhancements of the proposed framework are statistically robust, rather than artifacts of fortunate parameter initialization.

**Table 1:** Quantitative comparison of generation quality across different methods. The best results are highlighted in bold.

Method	Backbone	BLEU-4	ROUGE-L	BERTScore	Judge Score
GPT-4o	–	0.0541±0.0024	0.3349±0.012	0.6489±0.0023	2.2914±0.0492
Deepseek-r1	–	0.0230 ± 0.0012	0.1977 ± 0.0105	0.6385 ± 0.002	2.6342 ± 0.0644
Llama-3.1-8B	8B	0.0493 ± 0.0018	0.3154 ± 0.011	0.6380 ± 0.0026	1.812 ± 0.0475
Qwen3-4B (Zero-shot)	4B	0.0376 ± 0.0017	0.2722 ± 0.0143	0.6345 ± 0.0021	2.545 ± 0.0654
Qwen3-4B (Few-shot)	4B	0.1030 ± 0.0029	0.2974 ± 0.0146	0.6939 ± 0.0020	2.7301 ± 0.0714
Qwen3-4B (RAG)	4B	0.0602 ± 0.0043	0.2934 ± 0.0148	0.6512 ± 0.0032	2.6261 ± 0.0676
**SA-CoT (Ours)**	4B	**0.3031 ± 0.01**	**0.6405 ± 0.0252**	**0.7597 ± 0.0044**	**3.06 ± 0.0548**

* “Judge Score” refers to the LLM-as-a-Judge evaluation.

**Table 2:** Performance comparison on downstream tasks: Fault classification (discriminative) and vibration prediction (regression). The best results are highlighted in bold.

Method	Classification		Regression		
	Accuracy	Macro-F1	MAE	RMSE	*R* ^2^
MLP	0.6892±0.0001	0.6801±0	13.7345±0.0126	19.3678±0.0063	0.3606±0
LSTM	0.5268±0.0044	0.4260±0.0073	15.5112±0.0464	20.6220±0.0163	0.2751±0.0001
ResNet	0.702±0.0001	0.7033±0.0001	12.6030±0.1667	18.6884±0.0323	0.4047±0.0001
Qwen3-4B (Frozen)	0.6964±0.0129	0.6895±0.0011	11.7598±0.1599	18.2274±0.0229	0.4324±0.0001
**SA-CoT (Ours)**	**0.7321 ± 0.0185**	**0.7173 ± 0.0225**	**11.3132 ± 0.0774**	**17.5014 ± 0.0266**	**0.4767 ± 0.0001**

### Generative performance analysis

We first assess the model’s capacity to generate accurate, style-consistent diagnostic reports. The quantitative results, summarized in Table 1, reveal a significant performance inversion where our 4B-parameter SA-CoT model decisively outperforms prominent general Large Language Models.

To comprehensively evaluate the generative diagnostic capabilities of the proposed framework, our benchmarking strategy encompasses both cutting-edge proprietary models and representative open-source Small Language Models (SLMs). Specifically, we included widely adopted models operating at the 4B and 8B parameter scales (e.g., Qwen3-4B, Llama-3.1-8B). These SLMs serve as the primary comparative baselines, representing the current standard for resource-efficient architectures deployable in edge industrial environments.

Notably, SA-CoT achieves a BLEU-4 score of 0.3031, exceeding GPT-4o (0.0541) by a factor of approximately 5.6, and Deepseek-r1 (0.0230) by over 13 times. This massive disparity underscores the acute domain gap in industrial applications. General models typically generate verbose, safety-centric, but generic advice (e.g., advising contact with the manufacturer), whereas our fine-tuned model produces the precise, technical standard operating procedures required by field technicians (e.g., specifying relay checks in the safety loop). Deepseek-r1, despite its superior reasoning capabilities in mathematics and coding, underperforms in this context because its extensive reasoning chain format diverges fundamentally from the concise, directive style of valid maintenance logs.

Retrieval-Augmented Generation (RAG) is often considered a standard solution for domain adaptation. While the Qwen3-4B (RAG) baseline improves upon the Zero-shot setting, it lags significantly behind the SA-CoT approach (BLEU-4: 0.0602 vs. 0.3031). This suggests that while RAG successfully retrieves relevant knowledge chunks, such as error code definitions, it fails to internalize the diagnostic logic and the professional linguistic style of a senior technician. The high BERTScore of 0.7597 achieved by SA-CoT further confirms that the model generates responses that are semantically aligned with the ground-truth reasoning path, rather than merely matching keywords.

While standard n-gram metrics (BLEU, ROUGE) and semantic metrics (BERTScore) quantify text similarity, they are inherently insufficient to determine true diagnostic correctness in safety-critical industrial contexts. To rigorously evaluate the generative outputs, we designed a highly constrained LLM-as-a-Judge protocol (utilizing GPT-4o) to act as an “internal technical auditor.” The judge was explicitly prompted to evaluate the candidate responses based on three task-specific industrial criteria:

- **Source Authenticity (Weight: 40%):** The model is heavily penalized if it generates generic, textbook-style troubleshooting steps (a common form of LLM hallucination). Maximum scores are awarded only when the generated text accurately reflects the specific procedures and nomenclature of the proprietary internal manuals provided in the ground truth.
- **Diagnosis Actionability (Weight: 40%):** In field maintenance, technicians require decisive actions. The judge scores models higher for pinpointing specific, actionable hardware faults (e.g., “check the eccentric bolt of the tension wheel”) rather than providing vague, exhaustive lists of all theoretical possibilities.
- **Format Compliance (Weight: 20%):** Evaluating strict adherence to the predefined “Source-Analysis-Conclusion” Chain-of-Thought structure without extraneous conversational filler.

This multi-dimensional audit ensures that the generative evaluation perfectly aligns with real-world industrial safety standards.

In the LLM-as-a-Judge evaluation, SA-CoT secured the highest score of 3.06 out of 5.0, decisively surpassing the Few-shot baseline. This indicates a qualitative preference for the generated diagnostics, attributed to their professional tone, clarity, and actionable steps that effectively mimic the output of an experienced engineer.

A critical barrier to deploying LLMs in safety-critical industrial environments is the phenomenon of “hallucination”—generating plausible but unverified or incorrect technical procedures. In this study, the mitigation of hallucinations was quantitatively evaluated through our generative metrics. By utilizing the proprietary elevator maintenance manuals as the absolute ground truth, metrics such as BLEU-4 and ROUGE-L effectively served as inverse indicators of hallucination risk. As demonstrated in our results, the SA-CoT fine-tuned Qwen3-4B achieved a 5.6-fold improvement in BLEU-4 scores over the zero-shot GPT-4o baseline. Furthermore, our qualitative LLM-as-a-Judge evaluations specifically penalized fabricated error codes and unsanctioned physical interventions. The combination of the “Source-Analysis-Conclusion” CoT constraint and the high semantic fidelity (BERTScore) provides strong indirect evidence that the proposed framework effectively restricts the model’s generative freedom, thereby reducing the risk of hazardous hallucinations in offline evaluations. However, we explicitly acknowledge that these automated metrics cannot fully substitute for rigorous human evaluation, and the definitive validation of hallucination elimination in real-world maintenance scenarios requires future testing by certified domain experts.

### Performance on downstream tasks

Beyond the generative capabilities previously discussed, a pivotal contribution of this study is validating the capability of the model to decipher physical equipment states via textualized sensor embeddings. We extended our evaluation to discriminative and regression tasks, comparing our semantic approach against traditional numerical deep learning baselines. The comparative results are summarized in Table 2.

#### Fault classification (discriminative task).

To evaluate the model’s discriminative power, we utilized a proprietary dataset derived from real-world elevator monitoring systems. The input space comprises a six-dimensional feature vector representing critical telemetry data: speed, pressure, three-axis acceleration (x, y, z), and cabin temperature. The objective is to categorize the system state into distinct operational modes.

For this Fault Classification task, the raw telemetry data contained highly imbalanced fault occurrences. We filtered the dataset to focus on valid alarm classes (e.g., fault codes “6000019,” “1000005,” “1000004,” and “0” for normal operation) and applied a rigorous balancing strategy to construct the training set, preventing any majority-class bias from skewing the model’s predictions.

As illustrated in Table 2, our SA-CoT embedding strategy secured an Accuracy of 0.7321 and a Macro-F1 of 0.7173, establishing a clear margin over conventional architectures such as ResNet (0.702) and MLP (0.6892). This performance advantage stems from the fundamental difference in data processing mechanisms. Standard numerical models treat sensor readings as abstract sequences, often failing to detect subtle semantic dependencies between variables. For instance, a slight deviation in vertical acceleration occurring while the speed is zero might statistically resemble noise to a numerical model, yet physically it suggests a specific leveling anomaly. By translating these features into natural language descriptions, our framework activates the pre-trained world knowledge of the LLM to contextually interpret these signal combinations. Furthermore, the performance gain over the frozen, non-fine-tuned Qwen3-4B backbone (0.6964 to 0.7321) corroborates that our style-aware training successfully aligned the model’s latent space with the specific physical signatures of elevator mechanics.

#### Vibration magnitude prediction (regression task).

To further assess the model’s regression capabilities in continuous domains, we utilized the public “Elevator Failure Prediction” dataset. This corpus targets the predictive maintenance of elevator door systems, integrating electromechanical readings from ball bearing sensors with ambient environmental data (such as humidity). The primary objective is to predict the absolute magnitude of vibration, serving as a critical proxy for identifying early-stage mechanical wear.The transition from raw sensor logs to textually grounded inputs involved several strict preprocessing steps. First, missing feature values (NaNs) in the continuous sensor data (e.g., speed, pressure, acceleration) were imputed using a zero-filling strategy to preserve the structural dimensions of the sensor arrays without introducing artificial statistical noise. Conversely, for the regression task, any sample lacking a definitive continuous target value (vibration) was rigorously dropped from the dataset to maintain ground-truth integrity. Finally, the cleaned numerical features were subjected to our textualization module, mapping the sanitized floating-point arrays into structured natural language templates compatible with the LLM’s tokenizer.As detailed in Table 2, our proposed method demonstrates superior predictive precision, achieving the lowest Mean Absolute Error (11.04) and Root Mean Square Error (17.31) among all evaluated methods, alongside the highest *R*^2^ score of 0.49. A noteworthy observation is that the frozen Qwen3-4B backbone, even without specific fine-tuning, yielded an *R*^2^ score of 0.44, significantly outperforming specialized numerical baselines like LSTM (*R*^2^ 0.2751) and ResNet (*R*^2^ 0.4047). This phenomenon suggests that transformer-based language models possess robust inherent capabilities for modeling complex sequential dependencies, even when processing textualized numerical data. The application of our SA-CoT fine-tuning further elevated the *R*^2^ score by 0.05, validating that reasoning patterns acquired from textual maintenance logs effectively transfer to the numerical domain. Consequently, the SLM functions not merely as a text generator, but as a versatile, physics-aware feature extractor.

### Comparative analysis of reasoning mechanisms

**Fig 2:**
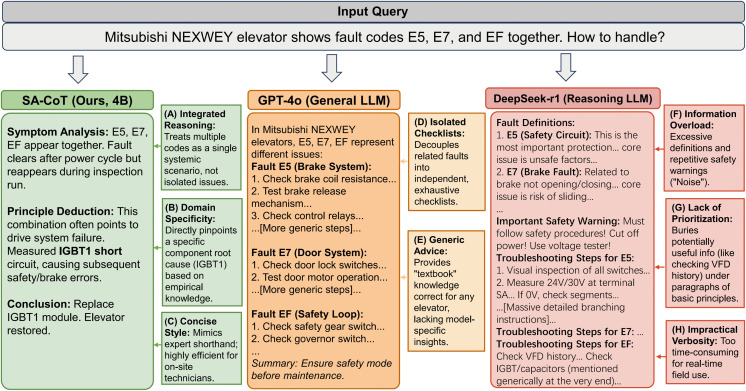
Visual comparison of diagnostic reasoning paths for a composite fault scenario (Mitsubishi NEXWEY: E5, E7, EF). While general models like GPT-4o and DeepSeek-r1 adopt a divergent approach, generating exhaustive but fragmented checklists, our SA-CoT model demonstrates convergent reasoning, identifying the causal link between faults and providing a concise, high-precision solution.

To provide an intuitive understanding of why our SA-CoT framework outperforms general-purpose baselines in industrial settings, we conducted a case study using a complex, multi-code failure scenario. Fig 2 visually contrasts the diagnostic reasoning paths of our model against GPT-4o, representing general LLMs, and DeepSeek-r1, representing reasoning-focused LLMs.

The selected query regarding the simultaneous appearance of E5, E7, and EF codes on a Mitsubishi NEXWEY elevator represents a typical cascade failure in elevator maintenance, where a single root cause triggers a chain reaction of alarms. As illustrated in the center and right channels of Fig 2, general models suffer from critical limitations in this context. GPT-4o adopts a divergent reasoning strategy, treating the three fault codes as independent variables. It decomposes the problem into three parallel, exhaustive checklists. While factually correct in isolation, this approach ignores the temporal and causal correlations between the codes, forcing the field technician to filter through generic definitions to locate the specific solution. Similarly, DeepSeek-r1, despite its strong logical capabilities, exhibits a low signal-to-noise ratio. It generates extensive preambles defining standard terms and repetitive safety warnings, effectively burying critical clues under paragraphs of basic principles. This verbosity significantly increases the cognitive load on operators during time-critical breakdown scenarios.

In stark contrast, our SA-CoT model demonstrates the efficacy of style-aware fine-tuning through convergent inference. Instead of listing all possible causes separately, it identifies the symptom cluster, inferring that the simultaneous appearance of E5, E7, and EF points dominantly to a drive system failure. This mimics the fast thinking pattern, often referred to as System 1, of a senior expert who relies on pattern recognition. Furthermore, the response style is strictly aligned with the Expert persona defined in our data augmentation strategy. By stripping away academic definitions and focusing solely on specific voltage measurement points and component replacement, specifically the IGBT1 module, the model reduces the troubleshooting workflow from navigating an exhaustive list of generic possibilities to performing a single critical check. This qualitative comparison validates that our framework does not merely memorize knowledge but learns to structure it, effectively bridging the gap between generic knowledge bases and practical industrial agents.

### Ablation study

To evaluate the individual contributions of our proposed modules, we performed an ablation study on the generative diagnostic task. We isolated the effects of the Style-Aware Augmentation and the Chain-of-Thought (CoT) reasoning constraints by comparing three training configurations:

- **SA-CoT (Ours):** The full framework utilizing both the style-aware augmented data and explicit CoT reasoning targets.
- **w/o Style:** The model trained solely on standard manual descriptions, excluding the style-augmented (Novice, Expert, and Noisy) samples.
- **w/o CoT:** The model trained on the full augmented dataset, but with intermediate reasoning steps removed from the target outputs, forcing a direct instruction-to-conclusion mapping.

As shown in Table 3, both components significantly impact the model’s diagnostic reliability. Removing the style augmentation (*w/o Style*) led to a consistent decline across all metrics, with the Judge Score dropping to 2.23. This suggests that training on diverse, noisy colloquialisms is necessary for real-world robustness, as field technicians frequently deviate from standard textbook terminology. Furthermore, the absence of CoT reasoning (*w/o CoT*) resulted in a much more severe performance penalty. The near-zero BLEU-4 (0.0041) and low Judge Score (1.73) indicate that without the structural constraint of step-by-step reasoning and source citation, the model struggles to anchor its outputs to the proprietary manuals. Instead, it tends to default to unverified, generic responses. This result supports the premise that explicit CoT constraints are vital for suppressing generative hallucinations in safety-critical industrial applications.

**Table 3:** Ablation study evaluating the impact of style-aware augmentation and CoT reasoning constraints. The best results are highlighted in bold.

Method	Backbone	BLEU-4	ROUGE-L	BERTScore	Judge Score
w/o Style	4B	0.2056	0.4034	0.7225	2.23
w/o CoT	4B	0.0041	0.0498	0.6244	1.73
**SA-CoT (Ours)**	4B	**0.3030**	**0.6412**	**0.7596**	**3.14**

* “Judge Score” refers to the LLM-as-a-Judge evaluation.

## Discussion

The results presented in this study demonstrate a paradigm shift in industrial fault diagnosis, advocating for a transition from opaque numerical models to interpretable semantic agents. Our findings challenge the prevailing assumption that larger parameter counts equate to better performance; the fine-tuned 4B-parameter SLM’s superiority over GPT-4o highlights that domain-specific alignment and knowledge density are the decisive factors for vertical industrial applications. Beyond text generation, this study establishes a robust methodological blueprint for integrating Large Language Models into complex physical environments.

### Robustness and numerical precision in the semantic-physical bridge

A core innovation of our framework is resolving the brittleness of traditional neural networks when faced with noisy, incomplete industrial telemetry. By abstracting raw high-frequency data into structured natural language, the textualization interface acts as a semantic filter, smoothing out transient mechanical noise and sensor dropouts to capture macroscopic degradation trends. Furthermore, we circumvent a fundamental bottleneck of LLMs: the loss of numerical precision caused by autoregressive tokenization. Instead of generating regression outputs token-by-token, we extract high-dimensional continuous embeddings from the SLM’s hidden layers, maintaining FP32/BF16 computational precision. This architecture explains the substantial quantitative leaps observed—such as a 75% error reduction over Support Vector Regression (SVR) and a 56% improvement over LSTMs in vibration prediction—proving that LLMs can function as highly effective, physics-aware feature extractors without sacrificing mathematical rigor.

### Safety, interpretability, and ethical accountability

Deploying generative AI within critical infrastructure necessitates stringent safeguards. Traditional data-driven models introduce unacceptable operational hazards due to their “black-box” nature. The SA-CoT framework structurally mitigates this risk through “Glass Box” interpretability. By forcing the model to explicitly cite verified manuals (*c*_1_) and articulate physical causality (*c*_2_) before suggesting interventions, it provides a transparent, auditable reasoning trail that neutralizes the danger of unverified hallucinations.Crucially, the system is governed by a strict “Defense-in-Depth” and Human-in-the-Loop (HITL) safety paradigm. The SLM functions exclusively as a decision-support advisor and is absolutely decoupled from the deterministic hardware fail-safes (e.g., Programmable Logic Controllers, mechanical limit switches). The AI possesses no authority to execute physical operational state changes, ensuring that the ultimate ethical, legal, and safety accountability remains strictly with certified human technicians.

### Cross-domain generalizability

While rigorously validated using a hybrid dataset of authentic proprietary elevator logs and open-source benchmarks, the proposed methodology is highly generalizable to the broader Industrial Internet of Things (IIoT). The underlying sensor textualization interface is fundamentally domain-agnostic; it can seamlessly map 1D time-series signals from other heavy electromechanical systems—such as wind turbine gearboxes or CNC machining spindles—without architectural modifications to the LLM backbone. By simply swapping the proprietary training corpus, the Style-Aware augmentation pipeline can automatically synthesize instruction datasets for new industries, offering a scalable blueprint for building autonomous, interpretable diagnostic agents across diverse smart manufacturing domains.

## Limitations and future work

While the SA-CoT framework demonstrates significant potential in bridging Small Language Models with industrial predictive maintenance, several limitations outline critical trajectories for our future research:

- **Dataset Scale and Cross-Domain Generalization:** The empirical evaluations rely on a dataset distilled from 11 proprietary manuals, inherently biasing the taxonomy toward specific elevator brands. Future work will focus on compiling larger, diverse datasets encompassing cross-brand legacy equipment. Additionally, we plan to scale the framework across diverse physical infrastructures beyond elevators to rigorously enhance its universal generalizability.
- **The Textualization Bottleneck for High-Frequency Data:** While textualization is a highly effective semantic bridge for low-frequency features, applying it to raw, ultra-high-frequency continuous signals (e.g., dense vibration waveforms) leads to exponential token expansion and the loss of fine-grained temporal dynamics. To address this, our primary objective is *direct multimodal integration*. We aim to develop native Multimodal Large Language Models (MLLMs) capable of directly ingesting raw time-series or spectrogram data alongside text, eliminating intermediate textualization bottlenecks.
- **Edge Hardware Deployment and Expert Validation:** The current study evaluates the framework within controlled, offline environments. Transitioning to safety-critical field deployment requires optimizing the 4B-parameter SLM via advanced quantization techniques (e.g., INT4/INT8) to reduce memory overhead and latency on resource-constrained industrial edge PCs. Crucially, this physical deployment must be accompanied by large-scale, double-blind qualitative evaluations conducted by certified human domain experts to rigorously verify diagnostic safety and actionability prior to commercial adoption.

## Conclusion

This study establishes a unified, interpretable framework for intelligent elevator maintenance, demonstrating that a lightweight Small Language Model (SLM) can effectively outperform massive, general-purpose foundation models in specialized industrial tasks. By employing a novel Style-Aware Chain-of-Thought (SA-CoT) fine-tuning strategy, we effectively grounded the model in proprietary knowledge, achieving a 5.6-fold improvement in BLEU-4 scores over GPT-4o while significantly reducing the risk of hazardous generative hallucinations. Furthermore, our approach successfully bridged the semantic-physical gap by textualizing continuous sensor data, allowing the SLM’s high-dimensional latent embeddings to outperform traditional numerical deep learning baselines in both fault classification and vibration prediction. Ultimately, these empirical findings highlight a significant shift in design philosophy: within vertical engineering domains, dense knowledge integration and strict structural alignment play a far more decisive role in diagnostic reliability than sheer parameter scale.
